# Supplementary material for: Dual role for Jumu in the control of hematopoietic progenitors in the Drosophila lymph gland
Source: eLife. 2017 Mar 28;6:e25094. doi: 10.7554/eLife.25094 (PMC5391210; doi:10.7554/eLife.25094)
Supplement: Supplementary file 1. — DOI: http://dx.doi.org/10.7554/eLife.25094.021 [file elife-25094-supp1.doc]

**Supplementary File 1**

**PCR primer sequences**

| **DNA fragment**  **(ChIP-PCR)** | ***Forward Primer (5' to 3')*** | ***Reverse Primer (5'to 3')*** |
| --- | --- | --- |
| -1993 to -1718 | ACACTTTCCATTTACCCGC | CAACATTCCGCTTTAGTATCC |
| -1763 to -1586 | AACGAATGGCTGTTTGTATG | ACACTCGCCCAACTACTCTG |
| -1406 to -1098 | TCGGAACCACCTGATGAT | AGGCGGATAGGAAGCAAG |
| -950 to -805 | TTCTACCATGCGGCCTAAC | TTTTTTATGTGCGTGTGTGC |
| -630 to -453 | ATCCCAAATAATGCCACAG | CGAAAATGTGTAGTTGACTGG |
| -454 to -258 | CGTATTGCATTTATTTCACAA | CCGCACACTGGTTCACAT |
| -255 to -105 | GAGGCAAAGAAACATTGATC | TTTAAGTGGTGAGAATAGGAGC |
| -46 to +235 | GCAGTCTGGTACGATAGAAATT | GCTCTTAGGTTATTTCTGGGA |
| **Mutagenesis** |  |  |
| -643 to +467 | AAAGGTACCCGGCAATGAAGAATCCCAAATAATG | AAACTCGAGTTCTATCCCGTTCCCGTGCC |
| M1 | CGATAGAAATTTTAGGTAAGCCACAGACAAC | GTTGTCTGTGGCTTACCTAAAATTTCTATCG |
| M2 | ACCAAACGCTATATCCATAGTTCTGTAG | CTACAGAACTATGGATATAGCGTTTGGT |
| M3 | GCAACACGTTTTAAGGTATACAACTACTAC | AGTAGTAGTTGTATACCTTAAAACGTGTTGC |
| ***target gene*** |  |  |
| *jumu* | GATAAGGAGTCAGTGCGTCG | AGTTAATGTCGACGCGAGC |
| *dmyc* | AAGAGTCGTGGCAAGAAGGT | GTATCGGCCTCATCAAAGC |
| *RpL32* | AGTCGGATCGATATGCTAAGCTGT | TAACCGATGTTGGGCATCAGATACT |
